# Resolution of phylogenetic position of Nigrofomitaceae within Hymenochaetales (Basidiomycota) and *Nigrofomes sinomelanoporus* sp. nov. (Nigrofomitaceae) from China

**DOI:** 10.3897/mycokeys.29.21250

**Published:** 2018-01-12

**Authors:** Li-Wei Zhou, Xue-Wei Wang, Josef Vlasák, Guang-Juan Ren

**Affiliations:** 1 Institute of Applied Ecology, Chinese Academy of Sciences, Shenyang 110016, China; 2 Institute of Plant Molecular Biology, Biology Centre of the Academy of Sciences of the Czech Republic, Branišovská 31, CZ37005 České Budějovice, Czech Republic; 3 Institute of Microbiology, Beijing Forestry University, Beijing 100083, China; 4 University of Chinese Academy of Sciences, Beijing 100049, China

**Keywords:** pantropical distribution, Polyporales, taxonomy, wood-inhabiting fungus

## Abstract

The family Nigrofomitaceae has been considered to be a member of Polyporales and a synonym of Polyporaceae for a long time. However, no molecular evidence supports this taxonomic opinion. For the first time, Nigrofomitaceae is included in a phylogenetic analysis, which shows that this family is separated from Polyporales and nested within Hymenochaetales as a distinct lineage from four well-known families, viz. Hymenochaetaceae, Neoantrodiellaceae, Oxyporaceae and Schizoporaceae. Therefore, Nigrofomitaceae is treated as the fifth family of Hymenochaetales. *Nigrofomes melanoporus*, the type species of Nigrofomitaceae, was considered to have a pantropical distribution. However, from both morphological and phylogenetic perspectives, the Chinese specimens labelled as *N. melanoporus* are found not to be conspecific with the specimens of *N. melanoporus* from Costa Rica, close to the type locality in Cuba. These Chinese specimens are thus described as a new species *Nigrofomes sinomelanoporus*. The species diversity of *Nigrofomes* in pantropical region is discussed.

## Introduction

Polyporales, accommodating about 2000 species, is one of the largest orders of wood-inhabiting fungi within Agaricomycetes, Basidiomycota (Kirk et al. 2008). The taxonomy of members of Polyporales has been extensively studied, resulting in the emergence of an enormous number of new genera and species worldwide (e.g. Cao et al. 2012, Miettinen and Rajchenberg 2012, Zhou and Dai 2012, Dai et al. 2014, Qin et al. 2016, Spirin et al. 2016, Wu et al. 2016). With the aid of molecular phylogeny, Binder et al. (2005) for the first time recovered four clades in Polyporales, viz. core polyporoid clade, antrodia clade, phlebioid clade and residual polyporoid clade. Later, an additional four lineages, viz. the family Fragiliporiaceae, the genus *Grifola* Gray, gelatoporia clade (or cinereomyces clade) and tyromyces clade emerged (Tomšovský et al. 2010, Miettinen and Larsson 2011, Miettinen and Rajchenberg 2012, Miettinen et al. 2012, Zhao et al. 2015). At the family level, 41 legitimate names are currently considered to belong to Polyporales (Binder et al. 2013, Zhao et al. 2015). Of these members, several families, including Nigrofomitaceae, have not yet been included in any phylogenetic analysis.

Nigrofomitaceae was erected to accommodate the monotypic genus *Nigrofomes* Murrill (Jülich 1981). This genus was typified by *Nigrofomes melanoporus* (Mont.) Murrill that was originally described from Cuba, tropical America (Murrill 1904). Recently, Hattori and Sotome (2013) combined *Trametes nigrivinea* Corner typified by a specimen from Papua New Guinea to *Nigrofomes* as *N. nigrivineus* (Corner) T. Hatt. & Sotome, bringing the members in this genus to two. Nigrofomitaceae was long treated as a synonym of Polyporaceae (Kirk et al. 2008, Ryvarden 2015), although the phylogenetic position of either species of Nigrofomitaceae remains unclear.

In the present study, Costa Rican and Chinese specimens of *Nigrofomes melanoporus* are analysed from a phylogenetic perspective for the first time and the phylogenetic affinity of Nigrofomitaceae is clarified. Moreover, the Chinese specimens labelled as *Nigrofomes melanoporus* are found not to be conspecific with the Costa Rican specimens of *N. melanoporus* and are herein described as a new species.

## Materials and methods

**Morphological examination.** The studied specimens were originally deposited at the herbarium of the Institute of Microbiology, Beijing Forestry University (BJFC) in China and the private herbarium of Josef Vlasák (JV) in the Czech Republic. In addition, the duplicates of all these specimens have been preserved at the herbarium of the Institute of Applied Ecology, Chinese Academy of Sciences (IFP) in China.

Macroscopic characters of the specimens were observed by the naked eye and also with the aid of a stereomicroscope. The microscopic procedure followed Kan et al. (2016). Specimen sections were mounted in Cotton Blue (CB), Melzer’s reagent (IKI) and 5% potassium hydroxide (KOH) and examined using a Nikon Eclipse 80i microscope at magnification up to 1000×. Measurements were taken in CB. The basidiospore size variation was presented by placing 5% of measurements from each end of the range in parentheses. Special colour terms followed Petersen (1996). Drawings were made with the aid of a drawing tube. The following abbreviations are used in the text: L = mean basidiospore length (arithmetic average of all measured basidiospores), W = mean basidiospore width (arithmetic average of all measured basidiospores), Q = variation in the L/W ratios between the specimens studied and n = number of basidiospores measured from a given number of specimens.

**Molecular sequencing.** Crude DNA was extracted from 0.02 to 0.2 g of dry basidiocarps of Costa Rican specimens using CTAB/NaCl followed by repeated extractions with chloroform and isopropanol precipitation. After purification and dilution, the DNA was used as a template for subsequent PCR amplifications. The primer pairs LR0R and LR7 (Vilgalys and Hester 1990) and ITS5 and ITS4 (White et al. 1990) were, respectively, selected for amplifying nLSU and ITS regions. The PCR procedure was as follows: initial denaturation at 95 °C for 5 min, followed by 35 cycles for nLSU region or 30 cycles for ITS region at 94 °C for 5 s, 55 °C for 15 s and 72 °C for 1 min and a final extension at 72 °C for 10 min. The PCR products were sequenced with the same primers in PCR amplifications in the Genomics Laboratory of Biology Centre, Academy of Sciences of the Czech Republic, České Budějovice, on an ABI 3730xl DNA analyser, using BigDye Terminator 3.1 kit.

The CTAB rapid plant genome extraction kit-DN14 (Aidlab Biotechnologies Co., Ltd, Beijing) was used to extract DNA from Chinese specimens according to the manufacturer’s instructions. The DNA was directly used as a template for PCR amplifications of the nLSU and ITS regions using the same primers as above. The PCR procedure was as follows for the nLSU region: initial denaturation at 94 °C for 1 min, followed by 34 cycles at 94 °C for 30 s, 50 °C for 1 min and 72 °C for 1.5 min and a final extension at 72 °C for 10 min, while for the ITS region: initial denaturation at 95 °C for 3 min, followed by 34 cycles at 94 °C for 40 s, 54 °C for 45 s and 72 °C for 1 min and a final extension at 72 °C for 10 min. The PCR products were sequenced with the same primers as those used for PCR at the Beijing Genomics Institute, China.

**Phylogenetic analysis.** The nLSU dataset, exploring the phylogenetic position of *Nigrofomes*, included sequences from species in Hymenochaetales and Polyporales as the ingroup and those in Thelephorales as the outgroup. To clarify the phylogenetic relationship between specimens of *Nigrofomes* from Costa Rica and China, the ITS dataset with *Oxyporus populinus* (Schumach.) Donk as an outgroup taxon focused on taxa closely related to *Nigrofomes* according to the topology inferred from the nLSU dataset. These two datasets were aligned using MAFFT 7.110 (Katoh and Standley 2013) with the g-ini-i option (Katoh et al. 2005). The resulting alignments were deposited in TreeBASE (http://www.treebase.org; accession number S21400). GTR + I + G and HKY + I + G were estimated as the best-fit evolutionary models for the resulting alignments from nLSU and ITS datasets, respectively, using jModelTest (Guindon and Gascuel 2003, Posada 2008). Following the corresponding models, the two alignments were subjected to phylogenetic analyses by maximum likelihood (ML) and Bayesian Inference (BI) methods. ML analysis was performed using raxmlGUI 1.2 (Silvestro and Michalak 2012, Stamatakis 2006) and bootstrap (BS) replicates were evaluated under the auto FC option (Pattengale et al. 2010). BI analysis was conducted using MrBayes 3.2 (Ronquist et al. 2012). Two independent runs, each including four chains of 10 million generations and starting from random trees, were employed. Trees were sampled every 1000^th^ generation. The first 25% of sampled trees was removed and the remaining trees were used to construct a 50% majority consensus tree and for calculating Bayesian posterior probabilities (BPPs). Chain convergence was judged using Tracer 1.5 (http://tree.bio.ed.ac.uk/software/tracer/). For each alignment, the ML and BI methods generated nearly congruent topologies and, thus, only the topologies generated from the ML method are presented along with the BS values and BPPs, respectively, above 50% and 0.8 simultaneously at the nodes.

## Results

**Figure 1.**
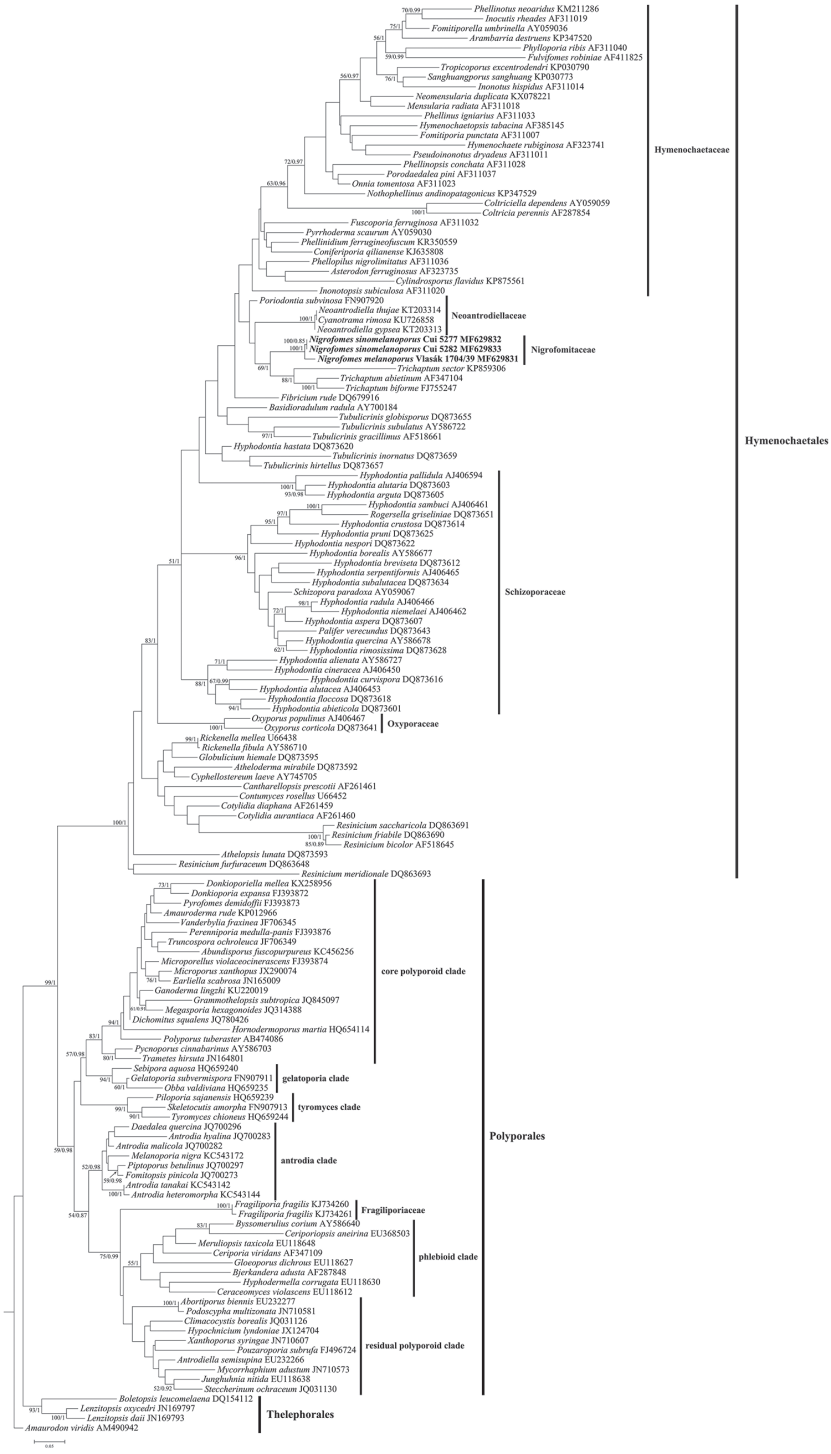
Phylogenetic position of Nigrofomitaceae inferred from the nLSU dataset. The topology is generated from the maximum likelihood analysis along with bootstrap values (above 50%) and Bayesian posterior probabilities (above 0.8), respectively, calculated from the maximum likelihood and Bayesian inference analyses at the nodes. Newly sequenced specimens are in boldface.

**Figure 2.**
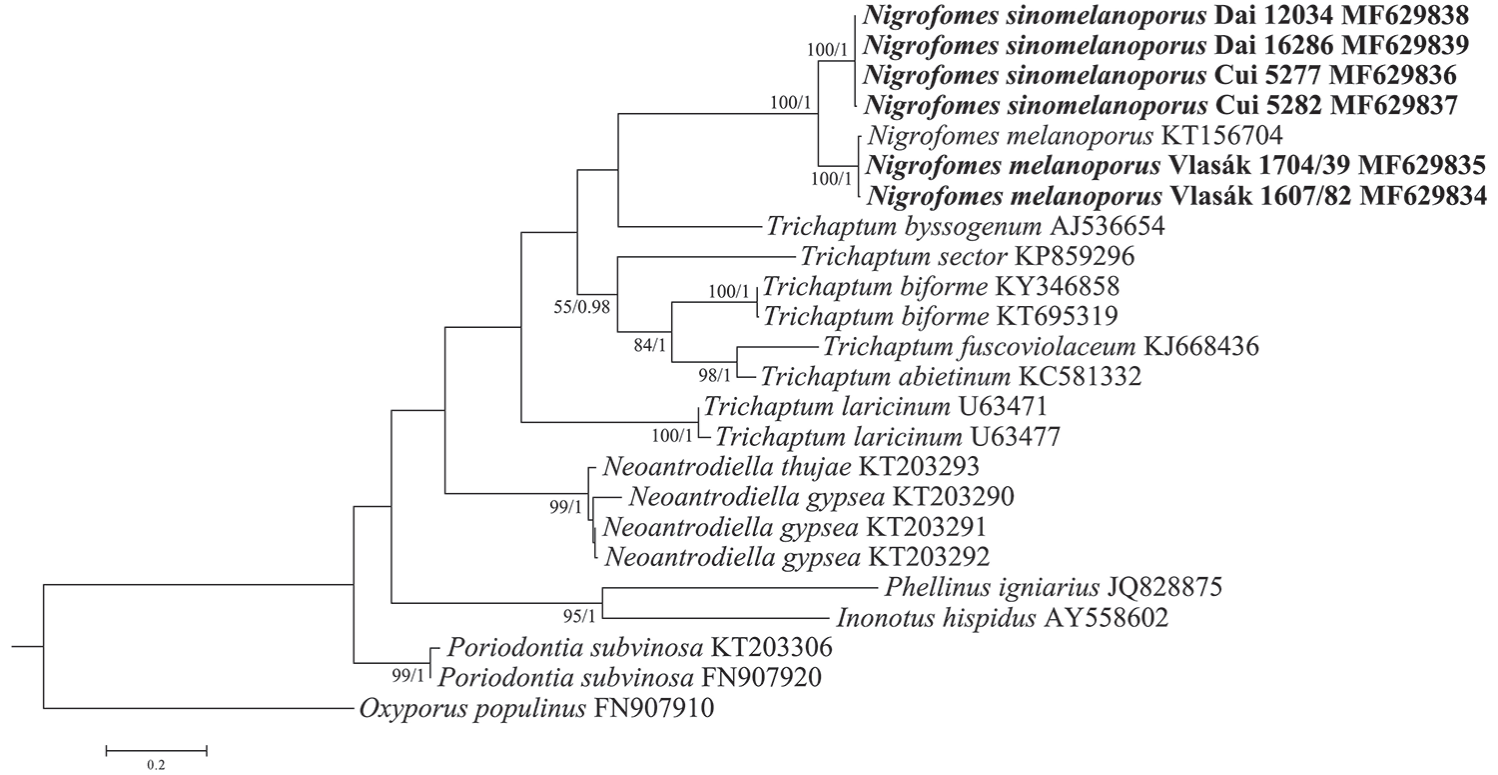
Phylogenetic relationship between the species of *Nigrofomes* inferred from the ITS dataset. The topology is generated from the maximum likelihood analysis along with bootstrap values (above 50%) and Bayesian posterior probabilities (above 0.8), respectively, calculated from the maximum likelihood and Bayesian inference analyses at the nodes. Newly sequenced specimens are in boldface.

Three nLSU and six ITS sequences were newly generated for this study and deposited in GenBank (http://www.ncbi.nlm.nih.gov/genbank; Figs 1, 2). The nLSU dataset, being composed of 147 sequences, resulted in an alignment of 956 characters. The BS search stopped after 400 replicates in the ML analysis, while all chains converged in the BI analysis, which was indicated by the effective sample sizes (ESSs) of all parameters above 1500 and the potential scale reduction factors (PSRFs) close to 1.000. In the nLSU-based phylogeny, Hymenochaetales (100% in ML and 1 in BI) and Polyporales (59% in ML and 0.98 in BI) were well separated; one Costa Rican and two Chinese specimens of *Nigrofomes* separated from each other (although not strongly supported in BI) but formed a distinct clade (100% in ML and 1 in BI) from widely accepted families within Hymenochaetales, viz. Hymenochaetaceae, Schizoporaceae, Oxyporaceae and Neoantrodiellaceae and also from other genera and species with uncertain phylogenetic position at the family level (Fig. 1).

The alignment, resulting from the ITS dataset of 24 sequences, comprised 1011 characters. After 250 replicates, the BS search stopped, while chain convergence was evidenced by the ESSs of all parameters above 5500 and the PSRFs equal to 1.000. The ITS-based phylogeny, focusing on *Nigrofomes* and related taxa within Hymenochaetales, shows that four Chinese and three Costa Rican specimens are clustered together but separated as two independent lineages, all with full statistical supports corresponding to their geographic origins (Fig. 2).

## Taxonomy

### Nigrofomes sinomelanoporus

Taxon classificationFungiPolyporalesNigrofomitaceae

L.W. Zhou sp. nov.

822281

Figs 3 , 4

#### Holotype.

CHINA. Hainan Province, Baisha County, Yinggeling National Nature Reserve, 17 Nov 2015, on dead standing angiosperm tree, Dai 16286 (BJFC 020373, isotype in IFP 019162).

#### Etymology.

*Sinomelanoporus* (Lat.): referring to the Chinese specimens similar to *Nigrofomes melanoporus*.

#### Description.

Basidiocarps perennial, effused-reflexed, pileate, solitary, without odour or taste when fresh, woody hard. Pilei triquetrous or applanate, fan-shaped to semicircular, projecting up to 7 cm long, 15 cm wide and 4 cm thick at base. Pileal surface dark brown to black, rimose with age, glabrous to tuberculate, distinctly concentrically zonate and sulcate with a distinct crust; margin sharp, black. Pore surface mouse-grey to vinaceous grey, glancing; sterile margin vinaceous brown, up to 5 mm wide; pores angular, 7–9 per mm; dissepiments thin, entire to slightly lacerated. Context vinaceous grey, woody hard, distinctly concentrically zonate, upside integrating with a distinct crust on the pileal surface, up to 1 cm thick. Tubes greyish brown to vinaceous grey, the fresh layer dark grey to black, woody hard, up to 3 cm long.

Hyphal system pseudodimitic; generative hyphae simple septate; all hyphae inamyloid, indextrinoid, acyanophilous; tissue unchanged in KOH. Context: generative hyphae hyaline to pale brown, slightly thick- to thick-walled with a wide lumen, rarely branched, frequently septate, 3–5 µm diam; skeletal-like hyphae dominant, pale brown, thick-walled with a wide lumen to subsolid, unbranched, occasionally septate, straight, more or less regularly arranged, 4.5–6 µm diam. Tubes: generative hyphae hyaline to pale brown, thin-to slightly thick-walled with a wide lumen, rarely branched, frequently septate, 2–5 µm diam; skeletal-like hyphae pale brown, thick-walled with a wide lumen to subsolid, unbranched, rarely septate, straight, more or less parallel along the tubes, 3.5–5 µm diam. Cystidia and cystidioles absent; basidia broadly ellipsoid to barrel-shaped, with four sterigmata and a simple septum at the base, 8–10 × 6.5–7.5 µm; basidioles in shape similar to basidia, but slightly smaller; basidiospores broadly ellipsoid to subglobose, hyaline, thin-walled, inamyloid, indextrinoid, acyanophilous, (4.5–)4.8–6(–6.7) × (3.8–)4–4.8(–5) µm, L = 5.18 µm, W = 4.27 µm, Q = 1.17–1.27 (n = 120/4).

**Figure 3.**
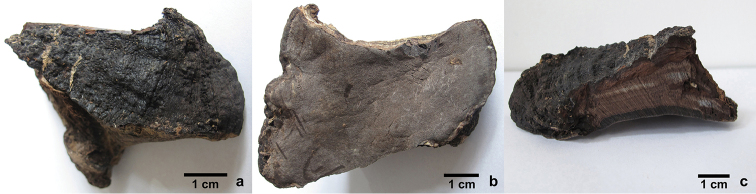
Basidiocarps of *Nigrofomes sinomelanoporus* (Dai 16286). **a** Pileal surface **b** Pore surface **c** A vertical section. Scale bars: 1 cm.

**Figure 4.**
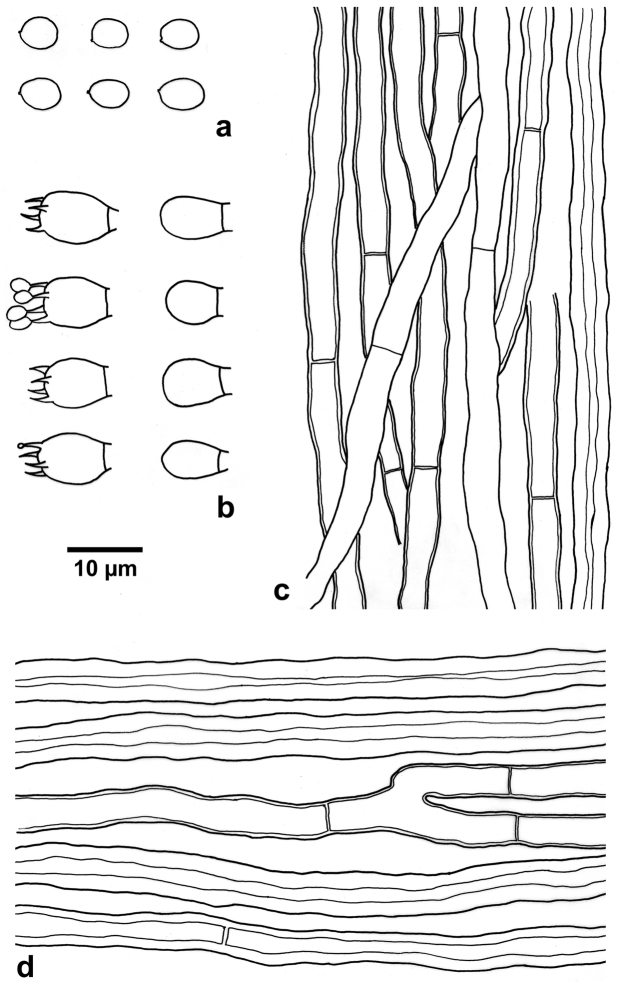
Microscopic structure of *Nigrofomes sinomelanoporus* (drawn from the holotype). **a** Basidiospores **b** Basidia and basidioles **c** Hyphae from trama **d** Hyphae from context. Scale bar: 10 µm.

#### Additional specimens (paratypes) studied.

CHINA. Hainan Province, Changjiang County, Bawangling National Nature Reserve, 25 Nov 2010, on dead standing tree of *Pentaphylax euryoides*, Dai 12034 (BJFC 009087, a duplicate in IFP 019163); Lingshui County, Diaoluoshan National Forest Park, 20 Nov 2007, on fallen angiosperm trunk, Cui 5277 (BJFC 003316, a duplicate in IFP 019164), Cui 5282 (BJFC 003321, a duplicate in IFP 019165).

#### Other specimens studied.

**Figure 5.**
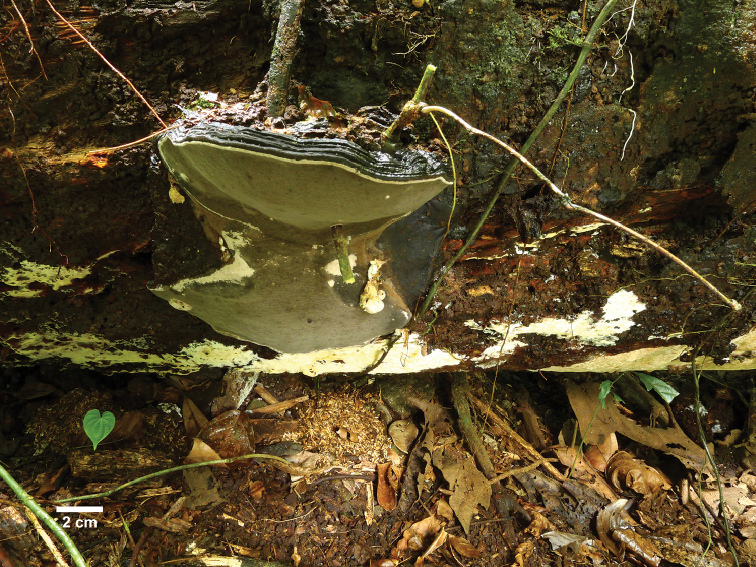
A basidiocarp of *Nigrofomes melanoporus* in situ (Vlasák 1704/39). Scale bar: 2 cm.

*Nigrofomes melanoporus*. COSTA RICA. Puntarenas Province, La Gamba Town, Piedras Blancas National Park, 20 Apr 2015, on fallen angiosperm trunk, Vlasák 1504/42 (JV, a duplicate in IFP 019166); Alajuela Province, Bijagua, Catarata Trail, 28 July 2016, on fallen angiosperm trunk, Vlasák 1607/82 (JV, a duplicate in IFP 019167); Puntarenas Province, Golfito Town, Playa Nicuesa Rainforest Lodge, 18 Apr 2017, on fallen angiosperm trunk, Vlasák 1704/39 (JV, a duplicate in IFP 019168; Fig. 5).

#### Note.

*Nigrofomes sinomelanoporus* differs by broadly ellipsoid to barrel-shaped basidia, absence of cystidia and larger basidiospores from *N. melanoporus*, which has clavate basidia, rare cystidia and smaller basidiospores (4–5 × 3–3.5 µm; Ryvarden 2015).

Regarding the hyphal system of *N. melanoporus*, Ryvarden (2015) recognised it as “probably dimitic” and Lowe (1966) as “dimitic”. They both mentioned the so-called skeletal hyphae are sometimes septate. According to the authors’ observations, there are two kinds of hyphae present in *N. melanoporus* and *N. sinomelanoporus* and one of them is frequently septate, whereas the other rarely or occasionally septate. When describing this kind of hyphal system, we prefer “pseudodimitic” to “dimitic” because genuine skeletal hyphae are generally defined as aseptate.

## Discussion

For the first time, Nigrofomitaceae was phylogenetically evidenced to separate from Polyporales and belong to Hymenochaetales (Fig. 1). Like Polyporales, Hymenochaetales is also an order mainly being composed of wood-inhabiting fungi. Four families, viz. Hymenochaetaceae, Schizoporaceae, Oxyporaceae and Neoantrodiellaceae were nested within Hymenochaetales in previous studies (Larsson et al. 2006, Zmitrovich and Malysheva 2014, Ariyawansa et al. 2015). However, the phylogenetic frame of this fungal order is not well resolved, which is indicated by the ambiguous phylogenetic position of many members of Hymenochaetales at the family level (fig. 1; Larsson et al. 2006, Miettinen and Larsson 2011, Ariyawansa et al. 2015). Even regarding the four accepted families, their circumscriptions are uncertain. For example, *Coltricia* Gray and *Coltriciella* Murrill, two genera morphologically and phylogenetically belonging to Hymenochaetaceae (Dai 2010, Ariyawansa et al. 2015), were excluded from Hymenochaetaceae according to the phylogenetic analysis inferred from nLSU and 5.8S regions (Larsson et al. 2006). Schizoporaceae has never yet been evidenced as monophyletic (Larsson et al. 2006). Oxyporaceae and Neoantrodiellaceae, two recently erected families, respectively based on one and four genera (Zmitrovich and Malysheva 2014, Ariyawansa et al. 2015), have not yet been fully explored. The current nLSU-based phylogeny did not resolve the circumscriptions of these four families similar to previous studies mentioned above, but did support the fact that Nigrofomitaceae, represented by the type genus *Nigrofomes*, occupied a distinct lineage outside these four well-known families and thus was considered to be the fifth family in Hymenochaetales (Fig. 1). In future, more comprehensive phylogenetic studies including many more representative samples and employing more loci will improve our understanding of the taxonomy of Hymenochaetales, which may result in the emergence of more taxonomic units at the family level.

*Nigrofomes melanoporus* was considered to have a pantropical distribution (Ryvarden 2015). However, after more careful morphological examination, the Chinese specimens previously labelled as *N. melanoporus* show distinct characters from the tropical American specimens. In the nLSU-based phylogeny (Fig. 1), the Chinese and Costa Rican specimens were separated as two lineages, but the clade of the Chinese specimens did not receive reliable support, whereas the phylogeny inferred from the ITS dataset including more samples of *Nigrofomes* strongly supported the Chinese specimens as an independent lineage (Fig. 2). Therefore, these Chinese specimens were newly described as *Nigrofomes sinomelanoporus* distinct from *N. melanoporus* from both morphological and phylogenetic perspectives. Hattori and Sotome (2013) distinguished *Nigrofomes nigrivineus* from *N. melanoporus* by the presence of clamp connections in the contextual generative hyphae of the former species. Only a single specimen of *N. nigrivineus* is known and no molecular sequence was provided (Hattori and Sotome 2013), which makes the position of this species ambiguous. However, both *N. sinomelanoporus* and *N. nigrivineus* indicate that the species diversity of *Nigrofomes* in pantropical regions could be higher than previously supposed.

## Supplementary Material

XML Treatment for Nigrofomes sinomelanoporus
